# Robust Self-Trapped Exciton Emission in Sb^3+^-Engineered Lead-Free Cs_4_SnBr_6_ Zero-Dimensional Perovskites

**DOI:** 10.3390/ma18235324

**Published:** 2025-11-26

**Authors:** Haixia Wu, Wendi Zhou, Rui Huang, Jie Song, Zhenxu Lin, Yi Zhang, Tianpei Qiu, Hongliang Li

**Affiliations:** School of Physics and Electronic Engineering, Hanshan Normal University, Chaozhou 521041, China; 20220063@hstc.edu.cn (H.W.); a54495597@126.com (W.Z.); rhuang@hstc.edu.cn (R.H.); songjie@hstc.edu.cn (J.S.); lzx2016@hstc.edu.cn (Z.L.); yee@hstc.edu.cn (Y.Z.); 202316001216@stu.hstc.edu.cn (T.Q.)

**Keywords:** Cs_4_SnBr_6_, zero-dimensional perovskites, self-trapped exciton, photoluminescence

## Abstract

Zero-dimensional (0D) tin halide perovskites have emerged as promising luminescent materials owing to their broadband emission, high quantum yield, and negligible self-absorption. Yet, their luminescence efficiency and stability remain insufficient for practical optoelectronic applications. Here, Sb^3+^ dopants are introduced into Cs_4_SnBr_6_ through a water-assisted wet ball milling strategy, resulting in bright and thermally robust emission. The doped materials exhibit pronounced self-trapped exciton (STE) luminescence centered at 525 nm with a broad full width at half maximum of 110 nm, a large Stokes shift of approximately ~1.3 eV, and a photoluminescence lifetime of ~0.8 µs. Remarkably, Sb^3+^ incorporation boosts the photoluminescence quantum yield (PLQY) up to 64% at room temperature while simultaneously improving thermal stability. Correlated spectroscopic analyses reveal that the Sb^3+^-induced lattice distortion of the [SnBr_6_]^4−^ octahedra strengthens electron–phonon interactions and elevates the STE binding energy, thereby stabilizing the excited states and suppressing nonradiative losses.

## 1. Introduction

Metal halide perovskites have emerged as a highly promising class of semiconductors for next-generation optoelectronic devices due to their remarkable intrinsic properties, including strong light absorption, high carrier mobilities, and exceptional photoluminescence quantum yields (PLQY) [1]. However, conventional three-dimensional (3D) lead halide perovskites face two critical challenges: poor intrinsic stability and the toxicity of Pb^2^⁺, which pose significant environmental and health concerns and severely impede their commercialization. This has spurred increasing interest in the development of lead-free alternatives with improved stability and low toxicity [2,3,4]. Among the emerging alternatives, zero-dimensional (0D) metal halide perovskites have attracted growing attention owing to their unique quantum confinement effect, broadband emission, high PLQY, and tunable photophysical properties [5,6,7,8]. These materials consist of isolated [MX_6_]^4−^ octahedral units spatially separated by A-site cations, endowing them with stronger exciton binding energies, improved environmental stability, and enhanced PL compared to their 3D counterparts [5,6,9,10,11,12,13,14]. In particular, 0D tin halide perovskites (Cs_4_SnBr_6_, X = Br or I) have emerged as environmentally benign emitters with high PLQY, due to their electronic structure similarity to lead-based analogues [12,14]. Nevertheless, Sn^2+^-based perovskites are inherently unstable—the high-energy 5s^2^ lone pair of Sn^2+^ is prone to oxidation to Sn^4^⁺, leading to structural degradation and PL quenching [14,15,16]. To mitigate this, Zhang et al. [14] used SnF_2_ as a stable tin precursor to prepare efficient Cs_4_SnBr_6_, while rapid thermal treatment (RTT) has also been shown to enhance self-trapped exciton (STE) emission by strengthening electron–phonon coupling and increasing exciton binding energy [17]. Despite these advances, achieving both high emission efficiency and robust thermal stability in 0D tin halide perovskites remains a critical challenge.

Ionic doping has proven to be an effective strategy to tune the optoelectronic properties of halide perovskites without fundamentally altering their host structure [18,19,20,21,22]. By introducing suitable impurity ions, it is possible to engineer band structures, modify the lattice, control defect density, and even generate new emissive centers. For instance, our previous work demonstrated that Mn^2^⁺ doping not only broadened the emission band of Cs_4_SnBr_6_ but also increased the PLQY to ~75% while enhancing thermal stability [23]. Among various dopants, Sb^3^⁺ ions have garnered particular interest owing to their unique ns^2^ electronic configuration [22,24,25,26,27]. Sb^3^⁺ can act as both an emissive center and a lattice modulator, enabling energy level tuning and nonradiative defect passivation [24,28]. Liu et al. [24] synthesized Sb^3+^-doped Cs_3_ZnCl_5_ phosphors with full-visible emission, while Zhou et al. [29] introduced Sb^3+^ into (C_8_H_22_N_2_Cl)_2_SnCl_6_ to passivate trap states and induce the formation of (SbCl_5_)^2−^ pyramidal units, which stabilized STE states and enhanced stability even under harsh conditions. Similarly, Jin et al. [28] demonstrated that Sb^3^⁺ doping in nonemissive Rb_4_CdCl_6_ softened the lattice and produced intense green emission at 525 nm with a PLQY of 70.2%. These studies highlight the great potential of Sb^3+^ doping in achieving efficient and stable emission in 0D perovskites. However, a systematic understanding of the effect of Sb^3+^ incorporation on the structural evolution, photophysics, and thermal stability of Cs_4_SnBr_6_ remains elusive.

In this work, we developed Sb^3+^-doped zero-dimensional Cs_4_SnBr_6_–SnF_2_ perovskites via a water-assisted wet ball milling method and systematically investigated the impact of Sb^3+^ incorporation on their crystal structure, PL, and thermal stability. The results reveal that appropriate Sb^3^⁺ doping leads to a dual enhancement of material properties, substantially boosting both the PLQY and thermal robustness of Cs_4_SnBr_6_. Comprehensive spectroscopic analyses further elucidate the underlying mechanism, showing that Sb^3+^ incorporation induces lattice distortion of [SnBr_6_]^4−^ octahedra, enhances exciton–phonon coupling, and stabilizes STE states, thereby enabling more efficient and thermally stable emission.

## 2. Experimental Section

### 2.1. Materials

Cesium bromide (CsBr, 99.9%, Aladdin, Shanghai, China), ammonium bromide (NH4Br, 99.99%, Aladdin, Shanghai, China), tin(II) fluoride (SnF2, 99.9%, Macklin, Shanghai, China), and antimony(III) bromide (SbBr_3_, 99.99%, Macklin, Shanghai, China) were employed as received without further purification. Deionized water served as the solvent during the milling process.

### 2.2. Synthesis of Sb^3+^-Doped Cs_4_SnBr_6_–SnF_2_ Powders

Sb^3+^-doped Cs_4_SnBr_6_–SnF_2_ perovskite powders were synthesized via a water-assisted wet ball milling route using an MSK-SFM-3-11 planetary ball mill (Hefei Kejing Material Technology Co., Ltd., Hefei, China). CsBr, SnF_2_, and NH_4_Br were used as the primary precursors, maintaining a fixed molar ratio of 4 mmol: 1 mmol: 2 mmol, respectively. The amount of Sb^3+^ was precisely adjusted to 0, 0.05, 0.1, 0.3, and 0.5 mmol to obtain a series of samples denoted as S–x (x = 0, 0.05, 0.1, 0.3, 0.5). In a typical synthesis, the precursor mixture was placed into a ball-milling jar along with 60 μL of deionized water and milled at 600 rpm for 30 min to ensure uniform mixing and reaction. The resulting slurry was subsequently dried at 60 °C under vacuum for 240 min. After cooling to ambient temperature, the dried solid was reintroduced into the jar and subjected to a second milling cycle at 600 rpm for another 30 min, yielding finely mixed Sb^3+^-doped Cs_4_SnBr_6_–SnF_2_ powders. The samples were not subjected to post-synthesis purification.

### 2.3. Characterization

The crystal structures of the synthesized powders were examined by X-ray diffraction (XRD) on a MiniFlex 600 diffractometer (Rigaku, Kyoto, Japan). Morphology and elemental distributions were investigated using field-emission scanning electron microscopy (FESEM, Hitachi SU5000, Tokyo, Japan) equipped with an energy-dispersive X-ray spectrometer (EDS, Bruker X-Flash Detector 630M, Karlsruhe, Germany). PL measurements, including steady-state spectra, temperature-dependent PL, and time-resolved PL decay, were carried out using an FLS1000 spectrofluorometer (Edinburgh Instruments, Livingstone, UK) coupled with a closed-cycle helium cryostat and temperature control system. The PLQYs were directly measured by using the same PL spectrometer (Edinburgh Instruments, Livingstone, UK) with an integrating sphere and excitation light at 340 nm. Power-dependent PL measurements were performed on a LabRAM HR Evolution high-resolution Raman spectrometer (HORIBA, Kyoto, Japan). To assess thermal stability, Raman measurements were combined with a Linkam THMS600 heating–cooling stage (Linkam Scientific Instruments Ltd., Redhill, UK), enabling in situ monitoring under controlled thermal environments. 

## 3. Results and Discussion

**Figure 1:**
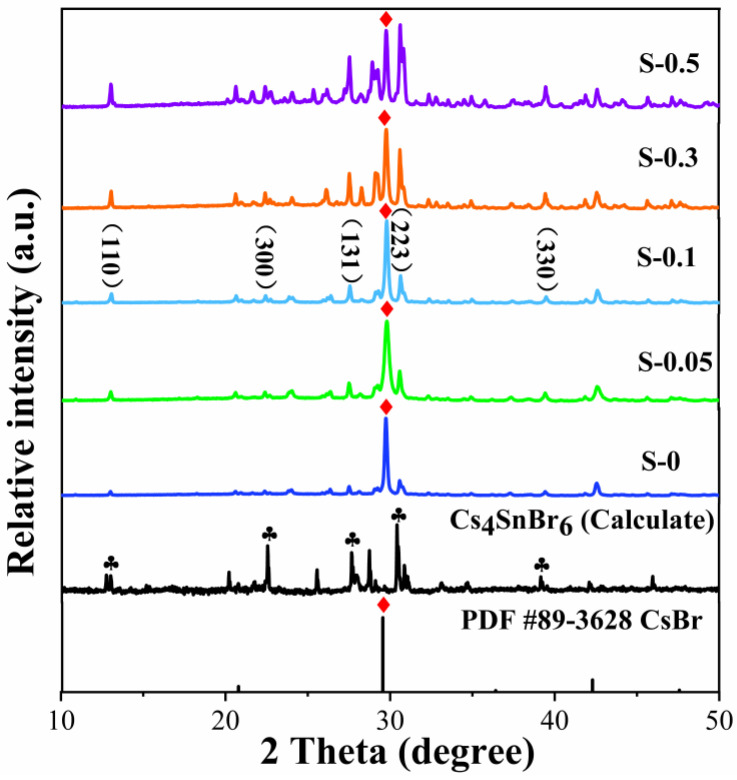
XRD patterns of Sb^3+^-doped Cs_4_SnBr_6_-SnF_2_ powders with various doping concentrations.

Figure 1 presents the XRD patterns of Cs_4_SnBr_6_-SnF_2_ powders containing different Sb^3+^ doping levels. The diffraction features reveal the coexistence of Cs_4_SnBr_6_ and CsBr phases, suggesting that a small portion of CsBr precursor remains unreacted during the solid-state milling synthesis. A distinct reflection near 29.7° corresponds to the CsBr phase (PDF #89-3628), whereas the characteristic peaks located at approximately 16.3°, 22.8°, 25.3°, 30.4°, and 33.8° are indexed to the (110), (300), (131), (223), and (330) planes of Cs_4_SnBr_6_, respectively. These reflections are in good agreement with previously reported patterns of SnF_2_-derived Cs_4_SnBr_6_ compounds [14]. As the Sb^3+^ concentration increases, the diffraction peak associated with the (223) plane gradually becomes narrower and more intense, indicating enhanced crystallinity and improved structural ordering within the perovskite lattice. Importantly, no additional peaks or shifts in the main reflections are detected, signifying that the introduction of Sb^3+^ does not alter the original phase composition. To further clarify the incorporation mechanism of Sb^3+^ in Cs_4_SnBr_6_, we considered the defect chemistry associated with heterovalent substitution. Because Sb^3+^ replaces Sn^2+^ on the B-site, each substitution event introduces one additional positive charge into the lattice. Even at the low doping levels employed here, charge neutrality must be maintained and is most plausibly accommodated by a very small concentration of charge-compensating point defects. For clarity, the doped compositions are therefore denoted as Cs_4_(Sn_1−x_Sb_x_)Br_6_, with the understanding that the lattice hosts a minor population of defect species required to balance the heterovalent substitution. This interpretation is consistent with our compositional analysis, which shows a gradual decrease in Sn content with increasing Sb loading and no detectable Cs deficiency. Furthermore, the pronounced PL quenching observed at higher Sb^3+^ concentrations (Figure 3) aligns with this scenario, as a larger density of charge-compensating defects would introduce additional nonradiative recombination pathways. Taken together, these results support a substitutional Sb^3+^-for-Sn^2+^ incorporation mechanism accompanied by a small defect population that becomes increasingly influential at higher doping levels.

**Figure 2:**
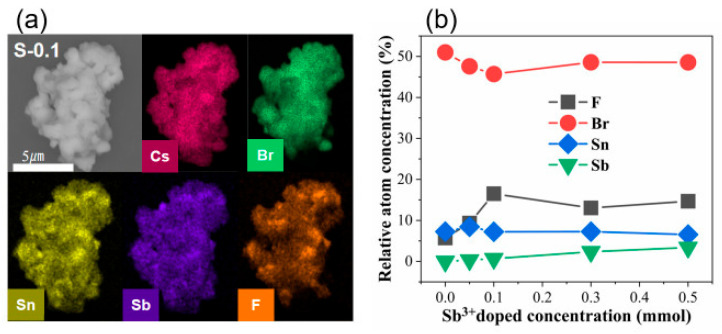
(**a**) SEM image and elemental mapping of Cs, Br, Sn, Sb, and F in the S-0.1 sample; (**b**) elemental composition evolution as a function of Sb^3+^ concentration.

Figure 2a shows the SEM micrograph of the S-0.1 sample along with the corresponding elemental mapping images for Cs, Br, Sn, Sb, and F. The uniform and continuous elemental signals across the entire particle region reveal that all constituent elements are evenly dispersed within the microstructure. Such homogeneous distribution also implies that introducing Sb^3+^ into the lattice does not lead to elemental segregation or local enrichment. The compositional evolution with increasing Sb^3+^ content is summarized in Figure 2b. A gradual decline in the relative atomic percentage of Sn is observed as the Sb^3+^ doping level increases. This inverse trend between Sb and Sn contents provides strong evidence that Sb^3+^ ions are incorporated into the host Cs_4_SnBr_6_ framework through substitution of Sn^2+^ lattice sites. Based on the EDS-derived elemental compositions shown in Figure 2b, the calculated B-site fractions Sb/(Sn + Sb) are 0.0388, 0.0837, 0.2449, and 0.3417 for the 0.05, 0.1, 0.3, and 0.5 mmol samples, respectively. These values confirm that the effective Sb incorporation remains well below the nominal precursor ratios, consistent with the limited solubility of Sb^3+^ in the Cs_4_SnBr_6_ lattice. Such low substitution levels account for the absence of measurable lattice-parameter changes in XRD across the doping series, indicating that Sb^3+^ induces only subtle local structural perturbations rather than large-scale lattice modification.

**Figure 3:**
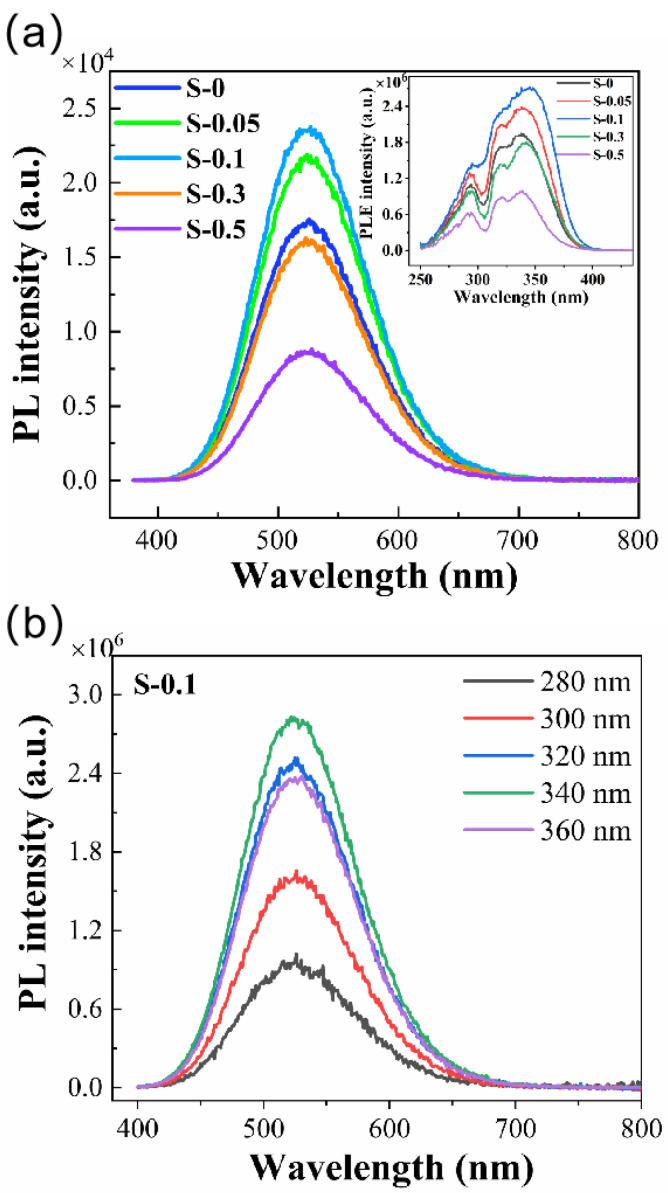
(**a**) PL emission spectra of Cs_4_SnBr_6_–SnF_2_ powders with different Sb^3+^ concentrations. The inset shows excitation spectra monitored at 525 nm; (**b**) PL spectra of the Cs_4_SnBr_6_ sample S-0.1 recorded under different excitation wavelengths.

**Figure 4:**
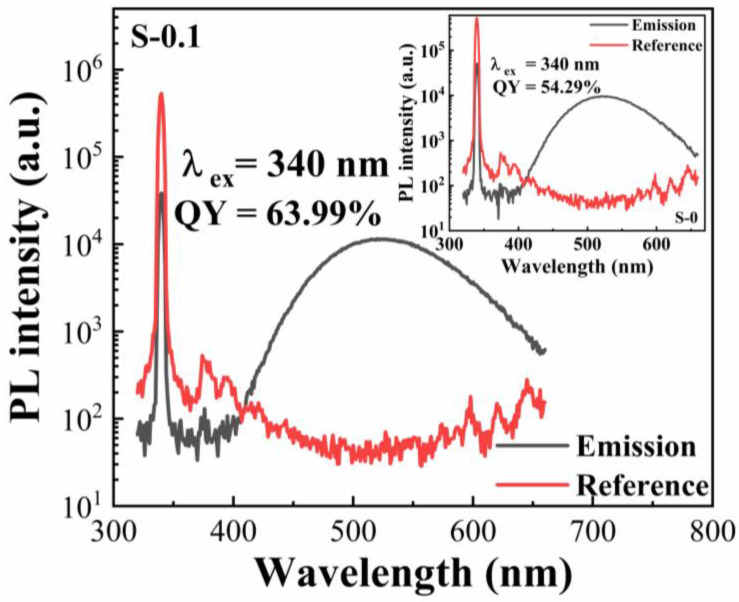
PLQY of the Cs_4_SnBr_6_ sample containing 0.1 mmol Sb^3+^. The inset shows the reference PLQY of the undoped Cs_4_SnBr_6_ sample. The PLQY is determined from the ratio of the number of emitted photons to the number of absorbed photons (Q = N_em_/N_abs_). The number of emitted photons (N_em_) is obtained from the integrated area of the spectrally corrected emission of the Sb^3+^-doped sample after subtracting that of the reference (N_em_ = A_em sample_-A_em ref_). The number of absorbed photons (N_abs_) is calculated from the difference in the integrated Rayleigh scattering peak areas of the reference and doped samples (N_abs_ = A_scat ref_-A_scat sample_).

**Figure 5:**
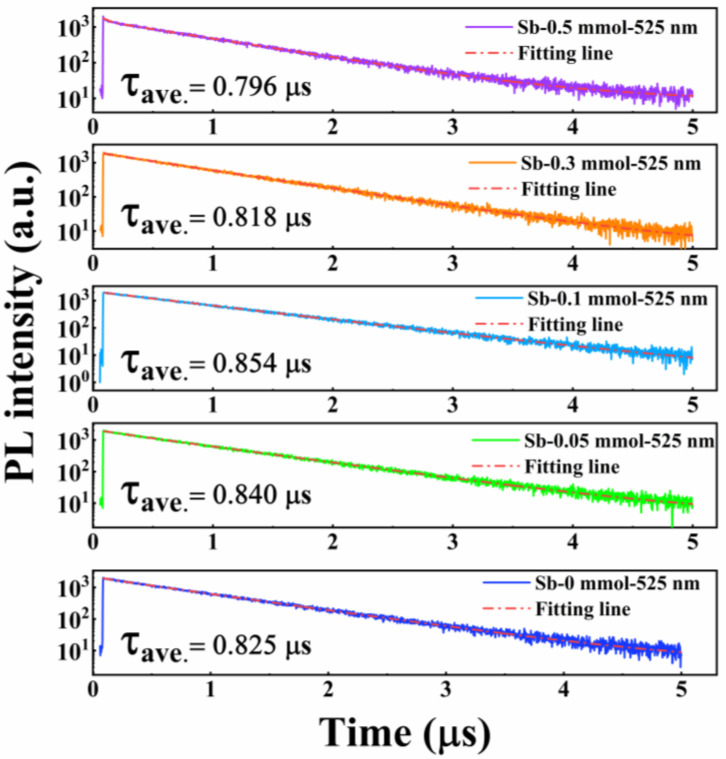
Time-resolved PL decay curves of Cs_4_SnBr_6_-SnF_2_ samples with various Sb^3+^ concentrations recorded at 525 nm. All measurements were performed under 375 nm excitation using laser pulses with a duration of 70 ps. The instrument response function of our setup is on the nanosecond scale.

The room-temperature PL emission spectra of Sb^3+^-doped powders with various doping levels are depicted in Figure 3a. Regardless of Sb^3+^ concentration, all samples display a characteristic broadband green emission centered near 525 nm with an FWHM of roughly 110 nm, a signature typically associated with STE recombination in 0D tin halide perovskites. As shown in the inset, when the emission wavelength is fixed at 525 nm, the optimal excitation wavelength consistently appears around 340 nm. This consistent excitation behavior across all compositions points to a common luminescent origin. The observed Stokes shift of ~1.3 eV aligns well with reported values for Cs_4_SnBr_6_ [9,12,14]. The effect of Sb^3+^ concentration on emission intensity is evident: the PL signal strengthens with increasing dopant content up to 0.1 mmol and then gradually weakens at higher concentrations. As shown in Figure 3b, the emission band shape remains unchanged when the excitation wavelength is varied from 280 to 360 nm. At the optimal level of 0.1 mmol, the PLQY is enhanced from 54.3% for the undoped sample to 64% (Figure 4). Moreover, the excitation and emission intensities follow similar variation trends, providing evidence that moderate Sb^3+^ incorporation can enrich the population of radiative excited states. Excessive Sb^3+^, however, may create additional nonradiative recombination pathways, thereby reducing the overall emission efficiency. It should also be noted that the minor CsBr phase detected in the samples is optically inactive in the visible region and thus does not contribute to the measured PL. The emission peak position in Figure 3 and the decay lifetimes in Figure 5 remain essentially unchanged across all doping concentrations, further confirming that the residual CsBr has no measurable influence on the intrinsic luminescence behavior of the Cs_4_SnBr_6_ host lattice.

To further elucidate the radiative recombination dynamics of Sb^3+^-doped Cs_4_SnBr_6_-SnF_2_ powders, TRPL measurements were carried out with the emission monitored at 525 nm. The resulting decay profiles for different Sb^3+^ concentrations are presented in Figure 5. Each curve can be well described by a biexponential function (Equation (1)), which captures both fast and slow recombination components [30]:

(1) $$I(t)=I_{0}+I_{1}exp(-t/\tau_{1})+I_{2}exp(-t/\tau_{2})$$

where *I*(*t*) is the emission intensity at time *t*, *I_0_* is a background constant, *I_i_* (*i* = 1, 2) are amplitude factors, and *τ_i_* denote the characteristic lifetimes. The decay traces reveal microsecond-scale dynamics with average lifetimes (*τ*_ave_) of roughly 0.8 μs for all samples. Such long-lived emission is a hallmark of STE-mediated recombination in zero-dimensional tin halide perovskites [11,12,31,32], corroborating the interpretation of the PL spectra. A clear concentration-dependent trend is observed: the average lifetime increases gradually at low Sb^3+^ doping levels, reaches a maximum at 0.1 mmol, and subsequently decreases with further doping. This behavior parallels the evolution of the PL intensity shown in Figure 3. The lifetime extension at moderate doping levels is likely associated with an increase in radiative STE population, whereas higher dopant concentrations promote nonradiative decay channels, indicative of concentration quenching.

**Figure 6:**
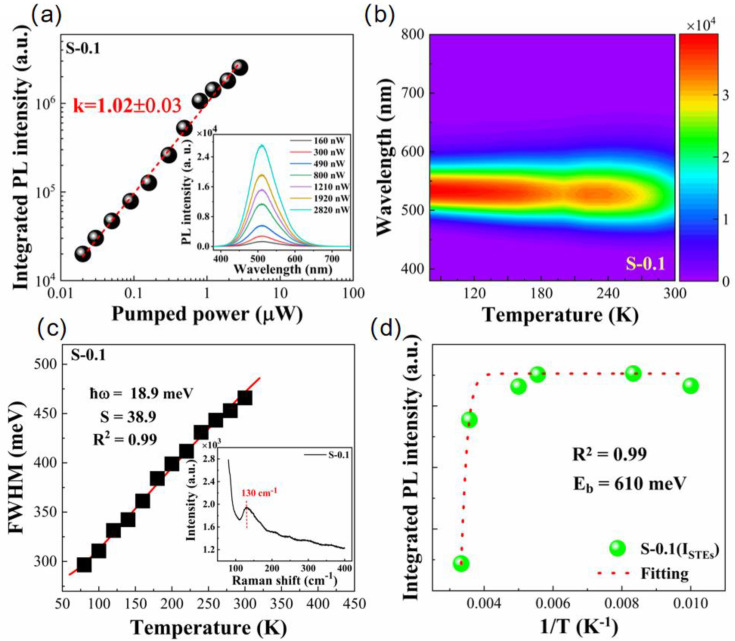
(**a**) Power-dependent PL intensity of S-0.1 powder with *k* = 1.02, consistent with excitonic emission. The inset shows corresponding PL spectra under different excitation powers; (**b**) temperature-dependent PL spectra (80–300 K) of S-0.1 powder; (**c**) temperature evolution of FWHM fitted with the Huang–Rhys model for S-0.1 powder. The inset displays the Raman spectrum showing Sn-Br stretching at 130 cm^−1^; (**d**) Arrhenius fitting of integrated PL intensity for S-0.1 powder yielding an exciton binding energy of 610 meV.

The power-dependent PL response of the S-0.1 powder reveals key information about its radiative recombination behavior. As illustrated in Figure 6a, when the excitation power is gradually increased from 160 to 2820 nW, the emission intensity rises proportionally, while the peak energy remains fixed. The absence of peak shifts suggests that the emission originates from an intrinsic transition rather than defect-assisted states. Over the entire excitation range, the integrated PL intensity follows a power-law dependence, described as follows:

(2) $$I=\eta I_{0}^{k}$$

where *I* is the integrated emission intensity, *P* represents the excitation power, and *k* is a characteristic exponent related to the dominant recombination channel. A best fit gives *k* = 1.02, consistent with exciton-mediated radiative recombination rather than defect- or band-edge-dominated processes [33,34]. When considered together with the large Stokes shift (~1.30 eV), broad emission (FWHM ≈ 110 nm), and microsecond-scale decay lifetime, these results support that the green emission arises from radiative relaxation of STEs within Jahn–Teller distorted [SnBr_6_]^4−^ units [10,12,32]. To probe the thermal response of this emission, variable-temperature PL spectra were recorded between 80 and 300 K (Figure 6b). The emission intensity becomes stronger at lower temperatures, which can be attributed to the suppression of thermally activated nonradiative recombination channels. Since the luminescence of STEs strongly depends on exciton binding strength, the temperature-dependent integrated PL intensity was analyzed using an Arrhenius model:

(3) $$I_{PL}(T)=\frac{I_{PL}(T_{0})}{1+\beta exp(-E_{b}/k_{B}T)}$$

where *I*_PL_(*T*_0_) is the PL intensity at 80 K, *β* is a prefactor related to nonradiative recombination, *k*_B_ is Boltzmann’s constant, and *E*_b_ denotes the activation energy for thermal quenching. The fitting yields *E*_b_ ≈ 610 meV (Figure 6d), substantially exceeding the value of the pristine sample [17], implying strong exciton localization and effective suppression of thermal detrapping in the doped system. The narrowing of the emission linewidth with decreasing temperature provides further insight into the coupling between electronic states and phonons. According to Stadler’s model, the temperature dependence of the FWHM is expressed as follows:

(4) $$FWHM(T)=2.36\sqrt{S}\hbar \omega_{phonon}\sqrt{{coth}_{2k_{B}T}^{\hbar \omega_{phonon}}}$$

where *S* is the Huang–Rhys factor, *ℏω* represents the phonon energy, and *k_B_* is Boltzmann’s constant. Fitting the data yields *S* ≈ 38.9 and *ℏω* ≈ 18.9 meV (Figure 6c), indicating pronounced electron–phonon interaction. This phonon energy (~152 cm^−1^) corresponds to the higher-frequency component of the Sn–Br vibrational manifold (inset of Figure 6c) [23,35], demonstrating that this vibrational mode is involved in STE formation. The enhanced STE emission observed at 0.1 mmol Sb^3+^ doping can therefore be attributed to the combined effect of stronger electron-phonon coupling and an increased exciton binding energy, which together promote efficient radiative recombination. At higher Sb^3+^ concentrations, however, excessive lattice distortion and defect formation introduce additional nonradiative channels, explaining the reduced PL intensity beyond the optimal doping level. It is also noteworthy that Sb^3+^ does not introduce dopant-centered emission; instead, it serves as an ns^2^-type lattice modulator that perturbs the [SnBr_6_]^4−^ octahedra and stabilizes intrinsic STE emission without altering its spectral characteristics. This behavior differs fundamentally from that of Mn^2+^-doped Cs_4_SnBr_6_, where Mn^2+^ not only modifies the lattice and enhances electron–phonon coupling but also acts as an activator center with its own radiative transition (Mn^2+ 4^T_1_ → ^6^A_1_), resulting in dual-band emission [23].

**Figure 7:**
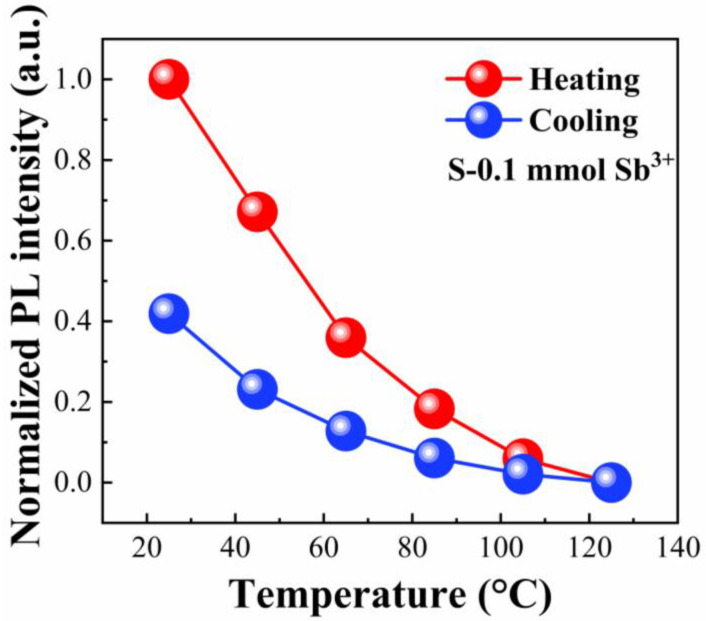
Normalized PL intensity of the S-0.1 sample recorded during heating–cooling cycles.

To assess the thermal resilience of the Sb^3+^-doped Cs_4_SnBr_6_ system, the integrated PL intensity was tracked during a full heating–cooling cycle. As illustrated in Figure 7, increasing the temperature from ambient conditions to 398 K leads to a continuous decrease in PL intensity, reflecting thermally activated quenching of the emissive states. Following a single heating–cooling cycle, approximately 40% of the original emission intensity is recovered. For comparison, undoped Cs_4_SnBr_6_ undergoes a far more pronounced degradation, retaining less than 10% of its initial PL intensity after similar thermal cycling [23]. This contrasting behavior points to a stabilization effect introduced by Sb^3+^ incorporation, which likely strengthens the self-trapped exciton states and suppresses competing nonradiative recombination pathways, thereby enhancing the thermal durability of the emission.

## 4. Conclusion

In this work, Sb^3+^ incorporation into Cs_4_SnBr_6_ zero-dimensional perovskites was achieved using a water-assisted wet ball-milling approach, enabling precise modulation of their photoluminescence behavior. A clear enhancement in both emission efficiency and thermal tolerance was observed, with the PLQY reaching 64% at the optimal doping level. Detailed investigations combining time-resolved PL, power-dependent PL, temperature-dependent measurements, and Raman analysis uncovered the underlying mechanism. The Sb^3+^ dopants induce lattice distortion of the [SnBr_6_]^4−^ framework, intensifying electron–phonon interactions and reinforcing the exciton binding energy of self-trapped excitons. These effects collectively promote radiative recombination while suppressing nonradiative loss pathways, resulting in more efficient and stable light emission. This strategy not only offers an effective means to manipulate the optical response of lead-free 0D tin halide perovskites but also sheds light on new design principles for robust luminescent materials. The strong and thermally robust green STE emission positions these phosphors as promising candidates for solid-state lighting and wide-gamut display technologies, particularly as environmentally benign, lead-free alternatives to traditional perovskite emitters. Moreover, the lead-free composition and low-temperature, scalable wet-milling synthesis route underscore their potential for sustainable photonic applications and large-scale industrial production.

## Data Availability

Data underlying the results presented in this paper are not publicly available at this time but may be obtained from the authors upon reasonable request.
